# Tropical Rain Forest Structure, Tree Growth and Dynamics along a 2700-m Elevational Transect in Costa Rica

**DOI:** 10.1371/journal.pone.0122905

**Published:** 2015-04-09

**Authors:** David B. Clark, Johanna Hurtado, Sassan S. Saatchi

**Affiliations:** 1 Department of Biology, University of Missouri, St. Louis, Missouri, United States of America; 2 La Selva Biological Station, Organization for Tropical Studies, Puerto Viejo de Sarapiquí, Heredia, Costa Rica; 3 Jet Propulsion Laboratory, California Institute of Technology, Pasadena, California, United States of America; Chinese Academy of Forestry, CHINA

## Abstract

Rapid biological changes are expected to occur on tropical elevational gradients as species migrate upslope or go extinct in the face of global warming. We established a series of 9 1-ha plots in old-growth tropical rainforest in Costa Rica along a 2700 m relief elevational gradient to carry out long-term monitoring of tropical rain forest structure, dynamics and tree growth. Within each plot we mapped, identified, and annually measured diameter for all woody individuals with stem diameters >10 cm for periods of 3-10 years. Wood species diversity peaked at 400-600 m and decreased substantially at higher elevations. Basal area and stem number varied by less than two-fold, with the exception of the 2800 m cloud forest summit, where basal area and stem number were approximately double that of lower sites. Canopy gaps extending to the forest floor accounted for <3% of microsites at all elevations. Height of highest crowns and the coefficient of variation of crown height both decreased with increasing elevation. Rates of turnover of individuals and of stand basal area decreased with elevation, but rates of diameter growth and stand basal area showed no simple relation to elevation. We discuss issues encountered in the design and implementation of this network of plots, including biased sampling, missing key meteorological and biomass data, and strategies for improving species-level research. Taking full advantage of the major research potential of tropical forest elevational transects will require sustaining and extending ground based studies, incorporation of new remotely-sensed data and data-acquisition platforms, and new funding models to support decadal research on these rapidly-changing systems.

## Introduction

A notable feature of the continental tropics is the very flat gradient in temperature decrease with increasing latitude [1]. In contrast, temperatures decrease much more rapidly over short horizontal distances with increasing elevation, generally ~0.6* C per hundred meters elevation increase [2], [3]. The biological consequences of steep elevational gradients juxtaposed with flat latitudinal temperature gradients in the tropics have stimulated research interest for decades, particularly since Janzen's seminal hypothesis on the effects of these gradients on species elevational ranges [4]. Recently tropical elevational gradients have excited interest as areas likely to be subject to rapid biological change due to global warming, and equally, as potential refugia for species fleeing upward to escape increasingly hotter temperatures at lower elevations [1], [5]. There is abundant evidence that upward migration of many species of plants and animals is already underway in both temperate and tropical areas [6], [7]. Tropical elevational transects are also important as major areas of high biodiversity, regional endemism, carbon storage, and locally as providers of key ecosystem services such as watershed protection [8], [9].

In this paper we describe the establishment of a network of forest inventory plots designed to study the long-term performance of tropical forest vegetation on a steep elevational transect. Forest structure and to a less extent dynamics along elevational transects have been described in several areas around the tropics (eg [5], [9], [10], [11], [12], [13], [14], [15], [16]. The research reported provides the first data on stand-level tree growth and dynamics along a steep elevational transect in Central America, adds information on the highest elevation forests that were not sampled in previous research, and extends the work of Heaney and Proctor [16] and Lieberman et al. [10] on forest physical and biological structure along this transect. In setting up this network we had to confront a number of issues common to sampling large complex landscapes, and we discuss how we addressed these challenges. As this research progressed we became increasingly aware of additional issues involved in studying long-term forest performance on tropical elevational transects. We discuss these theoretical and practical challenges, analyze the state of current research efforts to address these, and suggest future research needed to expand our current understanding of very small forest plots to large, heterogeneous tropical mountain forest landscapes.

## Methods

**Fig 1:**
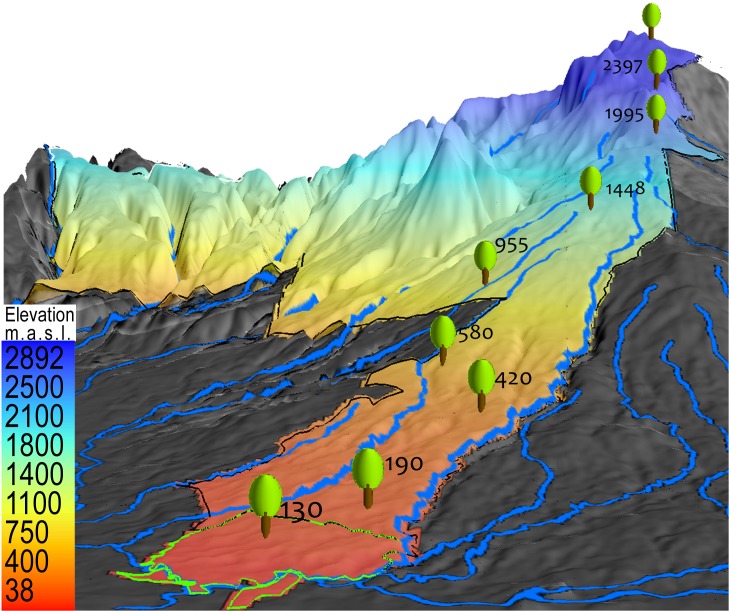
Plot locations. Location of the 9 1-ha forest inventory plots along the elevational gradient from 100 to 2800 masl on the eastern slopes of Volcán Barva, Costa Rica.

The study took place along an elevational gradient running from 130 m elevation at the La Selva Biological Station to 2830 m in Braulio Carrillo National Park on the Caribbean slopes of Volcán Barva in NE Costa Rica (Fig 1). Permission to carry out research in Costa Rica and Braulio Carrillo National Park was given by the Ministerio de Ambiente y Energía; permission to work at the La Selva Biological Station was granted by the Organization for Tropical Studies.

**Fig 2:**
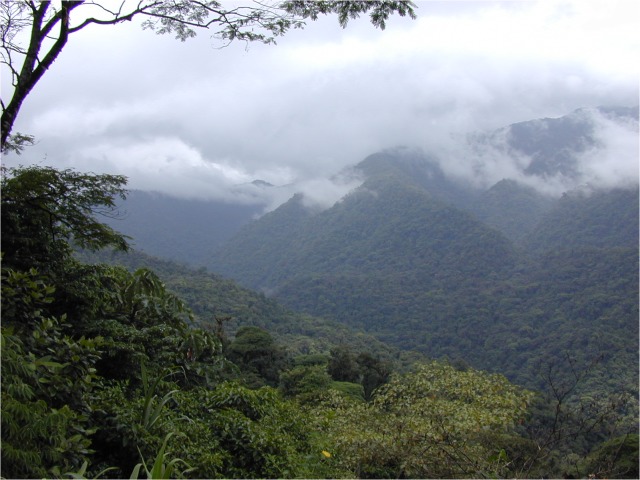
Study area terrain. Much of the study area in Braulio Carrillo National Park, Costa Rica, occurs on rugged roadless mountain slopes.

The study area is mostly extremely wet (≥4000 mm annual rainfall) old-growth forest, although there are significant areas of secondary forest resulting from natural disturbances such as landslides and earthquakes and from previous deforestation at some elevations. Previous work on the geology, soils, and soil litter chemistry is reviewed in [10]. The Volcán Barva transect begins in lowland Tropical Wet Forest [17], then rises through Tropical Premontane and Tropical Montane Forest to a small area of tropical montane cloud forest [18] at the summit (Table 1). The entire 50,000 ha area is permanently protected within the boundaries of Braulio Carrillo National Park and the La Selva Biological Station. This park is unique in Central America in protecting the entire elevational transect from lowlands to cloud forest. Apart from a highway on the eastern side of the Park, the area is roadless and extremely rugged (Fig 2), and lack of roads and very high rainfall were significant logistical issues.

**Table 1:** Plot descriptions.

Plot Name	Mean elevation	Latitude	Longitude	Life Zone	Mean Temperature	Mean Rainfall mm	N Years
100	129	10.42	-84.02	Tropical Wet Forest	25.0	3946	10
200	188	10.40	-84.04	Tropical Wet Forest	23.1	ND	9
400	417	10.35	-84.06	Tropical Wet, cool transition	22.3	ND	7
600	579	10.32	-84.05	Tropical Wet, cool transition	21.6	6057	7
1000	954	10.27	-84.06	Tropical Premontane Rain Forest	20.0	7451	4
1400	1448	10.24	-84.09	Tropical Premontane Rain Forest	17.8	ND	3
2000	1994	10.18	-84.11	Tropical Lower Montane Rain Forest	14.5	ND	5
2400	2397	10.16	-84.11	Tropical Lower Montane Rain Forest	12.1	ND	3
2800	2829	10.13	-84.11	Upper Montane Cloud Forest	10.4	ND	5

Plot names, mean elevation of the four corners from GPS data, latitude and longitude, life zone classification, mean temperature and rainfall, and number of years in which met data were collected. Life zone classifications for all plots but the 2800 m plot follow Hartshorn and Peralta [45], the 2800 m plot follows Scatena et al. [18]. ND = no data available.

Annual rainfall varies from ca. 4000 mm at the base to as much as 9000 mm at intermediate elevations, diminishing to ca. 3,000 mm at the summit. Temperature averages 25° C. in the lowlands, decreasing at roughly 0.54 ^o^ C/100 m elevation to ca. 10° C at the summit (Table 1).

Along this transect we established a network of 9 1-ha forest inventory plots (Table 1). Detailed protocols for site selection are explained in detail in S1 File. Briefly, the plots were designed to provide representative samples of old-growth forest to enable long-term study of forest composition and performance, stratified across the gradients of interest in the larger landscape, which in this case were primarily elevation, local topography, and stand history. Written protocols were developed to select, in a stratified random design, relatively flat old-growth sites with no signs of recent human disturbance and that contained no large stands of bamboo or permanent rivers or streams.

Square plots were surveyed to a planimetric area of 1.00 ha using slope-corrected distances and a surveying transit; for simplicity we refer to the plots by their elevation rounded to the nearest 100 m (Table 1). Within each plot all woody stems ≥10 cm diameter were mapped and tagged, identified to species (or morphospecies and collected) and censused annually for stem growth, mortality and recruitment using the vegetation monitoring protocols of the Conservational International Tropical Ecology Assessment and Monitoring (TEAM) Project (http://www.teamnetwork.org/en/protocols/bio/vegetation). Stem diameters were measured with a fabric diameter tape ±0.1 cm at 1.3 m above the ground or ~ 50 cm above basal irregularities such as buttresses, using ladders if necessary, at a permanently marked point of measurement (POM). For multiple-stemmed individuals all stems with valid POMs were measured. The raw forest inventory data and litterfall data can be freely downloaded from the TEAM Project data portal: http://www.teamnetwork.org/data/query.

We present these results in terms of number of individuals and statistics of diameters and basal areas. Given the wide range of elevation covered and the lack of local information on wood density and allometry for most species, we did not attempt to produce estimates of biomass, which would necessarily have been of unknown accuracy [19].

At the 100, 200, 400 and 600 m plots fine litterfall (leaves, flowers, fruits, wood litter <1 cm diameter) was monitored using 25 0.25 m^2^ standing basket traps per plot. Small woody litterfall (1–10 cm diameter) was monitored using 25 0.25 m^2^ staked areas on the forest floors. Litter traps were checked every two weeks and bulked per plot. Samples were over-dried at 65* C until weights stabilized to ±0.001 g for two consecutive weighings.

Canopy gap mapping was done on a 5 x 5 m grid in each 1 ha plot (N = 441 points/ha) using the methods described in Silva et al. [20]. A clinometer was used to establish verticality, the highest leaf above that point was determined (with binoculars if necessary), and heights up to 15 m were measured with a 15 m extending measuring pole by a two person team, one person observing and directing and one person raising the measuring pole. Heights >15 m were classified as one category. The complete canopy heights data set is given in S1 Dataset.

Crown heights to the highest leaf were measured using a Laser Technology Impulse laser ranger finder mounted on a tripod. Accuracy (approach to the directly measured "true" value) and precision (repeatability) in open conditions were on the order of decimeters. Precision in field conditions was several decimeters, while accuracy in field conditions is unknown, since direct measurement with an alternative method is generally not feasible. For trees with multiple stems and hence crowns we measured the height of each crown. The complete crown heights data set is given in S2 Dataset.

Temperature data were collected using microdataloggers (HOBO). The sensors recorded data every 30 minutes, from which we derived annual means. Precipitation data for the100 m and 600 m plots came from automatic weather stations of the Organization for Tropical Studies and were located within 3 km of these plots. Data for the 1000 m plot came from a manual rain gauge located at the Rara Avis Ecolodge, 2 km from the plot. The missing values of precipitation (ND in Table 1) are for plots with no weather station within 3 km.

## Results

### Biodiversity and life form patterns

Of the 5032 total individuals alive at the last census, 99% were identified to species or morphospecies. The sample included 83 identified families, 197 genera and 355 species or morphospecies, with 340 trees, 6 palms, 8 lianas and 1 tree fern. Species diversity (the total number of species in a plot census) peaked between 400–600 m, where it was 50–76% higher than lower elevation plots (Table 2). Above 600 m species diversity decreased steadily with elevation, dropping to only 19 species/ha in the 2800 m plot. Similar patterns of species diversity with elevation were reported by Liberman et al. [10], even though there were no plot locations in common with this research and eight plot elevations differed between that study and this one.

**Table 2:** Plant life forms by elevation.

Plot	Trees	Palms	Lianas	Tree ferns	All species	% Multi-stemmed individuals
**100**	89 (67.8)	4 (31.0)	4 (1.1)	0 (0.0)	97 (435)	0.2
**200**	93 (82.7)	5 (15.9)	3 (1.4)	0 (0.0)	101 (485)	0.4
**400**	143 (91.6)	4 (6.8)	4 (1.7)	0 (0.0)	151 (664)	0.3
**600**	156 (94.6)	3 (2.2)	5 (3.2)	0 (0.0)	164 (598)	1.5
**1000**	72 (84.3)	1 (13.6)	0 (0.5)	1 (1.6)	74 (427)	3.3
**1400**	57 (100.0)	0 (0.0)	0 (0.0)	0 (0.0)	57 (383)	2.3
**2000**	44 (96.0)	0 (0.0)	1 (0.4)	1 (3.6)	46 (445)	6.5
**2400**	35 (92.1)	1 (0.7)	0 (0.0)	1 (7.2)	37 (544)	7.9
**2800**	18 (99.8)	0 (0.0)	1 (0.2)	0 (0.0)	19 (1051)	10.3

Number of species by life form groups and as percentage of individuals (in parenthsis) in 1-ha plots in old growth over a 2700-m elevational gradient in Braulio Carrillo National Park, Costa Rica. Data are from the last census at each site (December 2013-May 2014). Total number of individuals per site is given in brackets in the All Species Column.

Abundance patterns among life forms were somewhat different (Table 2). Palms were a significant portion of the individuals in the stands below 1000 m, reaching their maximum in the lowest elevation site at 31% of individuals at the 100 m plot. Large lianas in contrast peaked in density at 600 m but the absolute numbers were relatively low at all sites. Although tree ferns occur across a broad elevational range on this transect, they were only found 3 plots between the 1000 and 2400 m elevation. Individuals with more than one trunk ≥10 cm diameter were rare below 500 m, but the frequency of such individuals increased with elevation, so that 10% of the individuals in the 2800 m site were multi-stemmed.

### Forest physical structure

There were two peaks in stem density (Table 3). Stem density was substantially higher at 400–600 m than at lower elevations or at higher elevations <2800 m. This pattern is also present in the data of Liberman et al. [10] (their Table 3). Stem density in the 2800 m plot was close to double the average of other plots. Basal area was also highest in the 2800 m plot, double to triple that in the other plots. In contrast, mean stem diameter varied only ca. 20% over the entire transect.

**Table 3:** Plot stand structure.

Plot	First and last census years	N censuses	Mean number of individuals ha^-1^	SEM	Mean basal area ha^-1^	SEM	Mean diameter cm	SEM
100	2003–2013	11	433	3.0	22.1	0.2	21.4	0.10
200	2004–2013	10	456	5.3	24.6	0.3	22.2	0.02
400	2006–2013	8	654	3.8	26.7	0.2	20.0	0.02
600	2006–2013	8	608	2.3	27.5	0.2	20.5	0.03
1000	2009–2013	5	415	3.0	20.2	0.1	21.4	0.08
1400	2010–2013	4	393	1.9	23.8	0.1	24.3	0.03
2000	2008–2013	6	478	8.3	26.7	0.3	22.3	0.09
2400	2010–2013	4	526	1.9	36.1	0.3	24.0	0.03
2800	2008–2013	6	1094	5.6	65.5	0.3	23.3	0.11

The number of individuals, plot basal area, and average stem size in 1-ha old-growth plots over an elevational transect on Volcán Barva, Costa Rica. Plots were censused annually. SEM = one standard error of the mean.

**Fig 3:**
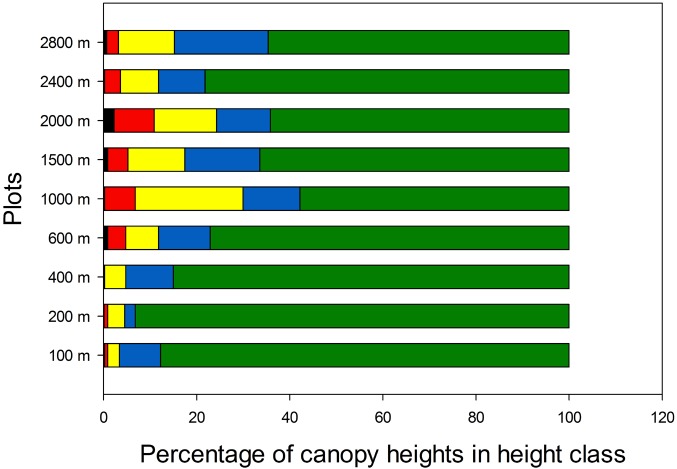
Canopy heights. Distribution of canopy heights along the elevational gradient. Percentages of measurements different height classes are coded left to right as: < 2 m, black; >2–5 m, red; >5–10 m, yellow; >10–15 m, blue; >15 m green. N = 441 at each site on a 5 x 5 m grid.

**Fig 4:**
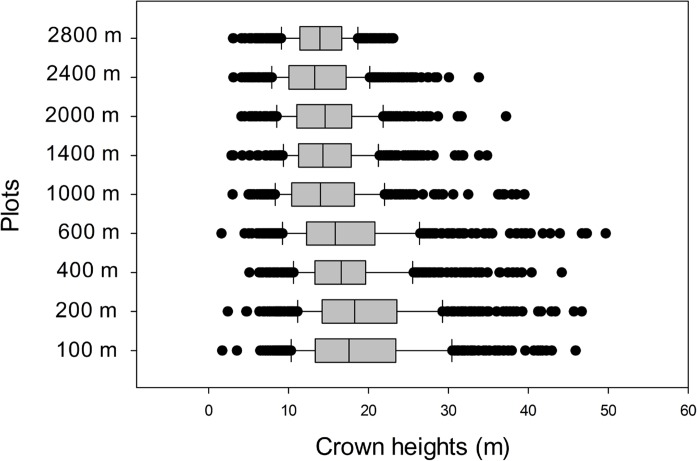
Crown heights. Box plots of crown heights along the elevational transects. Left and right vertical lines are 10th and 90th percentiles, box encloses 25–75 percentiles with the median a vertical line.

Canopy gaps extending to ≤2 m from forest floor ("Brokaw gaps", Brokaw [21] were uncommon at all elevations (Fig 3). Canopy and subcanopy structure on the other hand varied considerably between lower and higher elevation plots. Below 1000 m most canopy height measurements were in the ≥15 m class, whereas from 1000 m elevation upwards canopy heights in the 2–15 m range were much more frequent. The 2400 m plot was anomalous in having a canopy structure very similar to lowland plots, with 78% of the canopy height measurements in the >15 m category. Crown heights were highest in the lowlands (Fig 4). As elevation increased, the maximum, mean, median, range and coefficient of variation of crown heights decreased. The distribution of crown heights at 2800 m was notably compressed, as evidenced by a difference of only 9 m between the median and maximum heights, compared to 26–34 m difference in below 1400 m.

### Dynamics and productivity

Individuals' diameter growth rates were highest in the lowlands, falling to about half those values at 2800 m (Table 4). Stand-level basal area productivity however, which is a function of both diameter growth rates and the number of individuals, showed a very different pattern. Lower rates of stem diameter growth at mid- and upper elevations were offset by higher stem densities, so that plot basal area increment was highest in the two highest elevation plots and peaked at 2800 m. We did not attempt to estimate woody productivity, because we do not have site-specific data on wood density or allometric variation in the relations among diameter, height and measured biomass over the elevation range. However, the high basal area addition of the highest elevation plots raises the interesting possibility that above-ground wood production may not vary greatly over this 2700 m range, since the much larger number of stems in these plots to some extent offsets the lower mean tree heights and slower diameter growth rates.

**Table 4:** Plot productivity.

**Plot**	**Dates**	**Mean fine litterfall Mg ha** ^-1^ **dy** ^-1^	**SEM**	**Mean woody litterfall Mg ha** ^-1^ **dy** ^-1^	**SEM**	**Mean plot BAI m** ^2^ **ha** ^-1^ **yr** ^-1^	**SEM**	**Mean diameter growth cm yr** ^-1^	**SEM**
100	2003–2013	0.026	0.001	0.00222	0.00013	0.50	0.02	0.31	0.010
200	2004–2013	0.030	0.001	0.00326	0.00021	0.55	0.01	0.32	0.015
400	2006–2013	0.028	0.001	0.00341	0.00020	0.45	0.02	0.22	0.012
600	2006–2013	0.027	0.002	0.00361	0.00036	0.47	0.01	0.21	0.007
1000	2009–2013					0.34	0.02	0.24	0.016
1400	2010–2013					0.44	0.01	0.30	0.012
2000	2008–2013					0.31	0.05	0.15	0.015
2400	2010–2013					0.64	0.03	0.27	0.010
2800	2008–2013					0.84	0.04	0.15	0.004

Rates of litterfall (dry weight, leaf, reproductive, and twigs <1 cm diameter), plot basal area increment (summed basal area increment of all individuals surviving a census interval) and mean diameter growth (cm yr^-1^) in1-ha plots in old growth along an elevational transect on Volcán Barva, Costa Rica. Litterfall at the four lowest elevation sites was taken biweekly from the first data of census through 2010. SEM = 1 standard error of the mean.

Diameter growth rates and basal area productivity in the 2000 and 2400 m plots was anomalous for unknown reasons. In the 2000 m plot basal area addition and mean diameter growth rates were considerably lower than in plots above and below, while in the 2400 m plot both rates were higher than expected by elevation and stem number (Table 4). Over the 450 m elevational gradient covered by the litterfall plots, there was no elevational trend in total litterfall. Total litterfall averaged about 11 Mg/ha/yr, with leaf litterfall an order of magnitude larger than fine woody litterfall (Table 4).

Rates of individuals' mortality, recruitment and turnover showed mixed relations to elevation. Over the period encompassed by this study (3–10 years depending on the site), the sites between 1000 and 2000 m had higher mortality rates than higher or lower elevation sites (Table 5). Rates of individual recruitment and turnover were however negatively related to elevation (adjusted r^2^ 0.55 and 0.41 respectively, both P<0.05).

**Table 5:** Plot dynamics.

Elevation	N	Mean Mort	SEM	Min	Max	Mean Rec	SEM	Min	Max	MeanTO	SEM	Min	Max
**100**	10	0.0172	0.0021	0.0097	0.0271	0.0284	0.0039	0.0096	0.0460	0.0228	0.0020	0.0145	0.0308
**200**	9	0.0168	0.0024	0.0025	0.0252	0.0353	0.0078	0.0128	0.0949	0.0260	0.0044	0.0149	0.0589
**400**	7	0.0139	0.0019	0.0055	0.0197	0.0201	0.0023	0.0137	0.0315	0.0170	0.0013	0.0129	0.0219
**600**	7	0.0199	0.0032	0.0077	0.0313	0.0205	0.0023	0.0093	0.0288	0.0202	0.0021	0.0085	0.0255
**1000**	4	0.0309	0.0063	0.0150	0.0435	0.0215	0.0039	0.0142	0.0326	0.0262	0.0018	0.0236	0.0314
**1400**	3	0.0269	0.0079	0.0163	0.0424	0.0215	0.0013	0.0195	0.0239	0.0242	0.0045	0.0186	0.0332
**2000**	5	0.0298	0.0078	0.0075	0.0564	0.0085	0.0026	0.0019	0.0181	0.0191	0.0036	0.0075	0.0292
**2400**	3	0.0102	0.0009	0.0091	0.0121	0.0215	0.0023	0.0171	0.0251	0.0158	0.0013	0.0133	0.0171
**2800**	5	0.0111	0.0026	0.0049	0.0200	0.0034	0.0008	0.0022	0.0063	0.0073	0.0016	0.0037	0.0131

Rates of individuals' mortality, recruitment and turnover in old-growth tropical rain forest along an elevational gradient in Costa Rica. Mort = mortality, Rec = recruitment, TO = turnover, SEM = one standard error of the mean, min = minimum, max = maximum. Census dates are given in Table 3.

**Fig 5:**
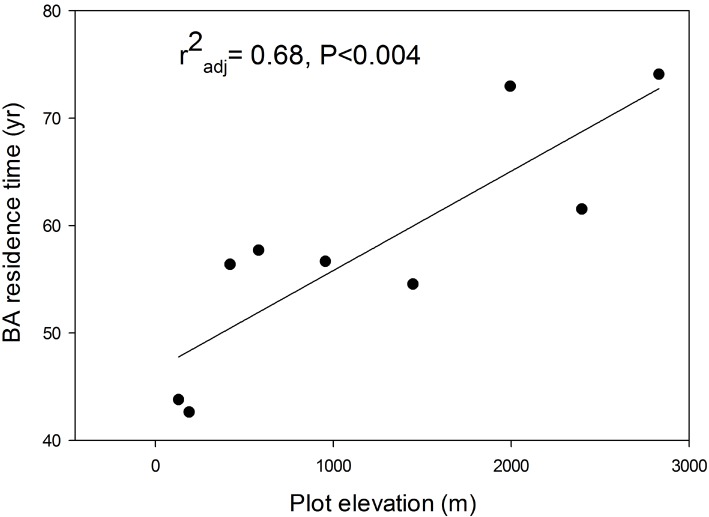
Basal area residence times. Rates of basal area residence time along an elevational transect in Costa Rican rain forest. Basal area residence time was calculated as the mean plot basal area over the interval divided by the mean rate of plot basal area addition (stocks divided by flux).

In general metrics of growth are less variable indicators of plot dynamics than vital rates like mortality and recruitment. Rates of individuals' mortality and recruitment are based on small sample sizes for plots at this spatial and temporal scale (one hectare plots sampled annually for 3–10 yr), and are therefore subject to significant stochastic variation as well as any variation in response to forcing factors. Growth rates however are always based on every surviving individual; in these data growth samples sizes are approximately two orders of magnitude larger than samples of dead or recruited individuals, so growth metrics are less subject to stochastic variation. In this study the coefficients of variation of diameter growth rates averaged 11% while those for mortality averaged 42%. Plot dynamics based on growth metrics showed a clear pattern of declining dynamics with elevation as well as dynamics consistent with old-growth forests. Basal area residence time (mean plot basal area/mean plot basal area increment) increased significantly with elevation (Fig 5). As expected in old-growth stands, stands with faster growth rates had lower basal area residence times (adjusted r^2^ = 0.789 P<0.001).

### Temporal patterns in structure and process

There were no consistent trends in changes in forest structure over time across the elevational gradient (Table 6). The lowest elevation plots gained basal area and individuals while the three plots from 1000–2000 m lost basal area and individuals, and results at the other plots were mixed. Data on mortality and recruitment varied considerably from year to year, and at most sites the range of variation was the same order of magnitude as the average value (Table 5). Given this variation and the relatively few data points at each site (3–10), it is not surprising we saw no general temporal trends in mortality and recruitment. Annual diameter growth rate in lowland forests of this area has been shown to be tightly coupled to interannual variation in rainfall and temperature [22]. While diameter growth rates varied from year to year, we found no consistent temporal trends in these short time series data among sites.

**Table 6:** Temporal trends in structure.

Plot	N censuses	% change in basal area	% change in number of individuals
100	11	6.5	8.7
200	10	10.8	12.1
400	8	4.2	3.9
600	8	-2.5	0.3
1000	5	-2.0	-1.9
1400	4	-1.8	-1.5
2000	6	-5.6	-10.4
2400	4	3.5	1.7
2800	6	2.5	-2.5

Changes in plot structure of time in 9 1-ha plots along a 2700-m elelvational gradient in Costa Rican rain forest. Censuses were conducted annually.

## Discussion

### Results synthesis and comparison with other tropical elevational transects

The 2700 m elevational transect spans a gradient of about 15* C in mean annual temperature and at least a two-fold variation in annual precipitation. In spite of this variation, the forests studied were fairly similar in physical structure and dynamics, with the exception of the summit cloud forest. Excepting the cloud forest, basal area, stem number, and diameter growth rates varied by a factor of two or less, while mortality and recruitment varied somewhat more. The cloud forest was completely distinct, with the highest stem number, basal area, and plot basal area increment, very low turnover, and greatly reduced total species number and individual growth rates.

A number of studies have described tropical rain forest structure, and in fewer cases function, across elevational transects in the tropics [5], [9], [10], [11], [12], [13], [14], [15]. It is not surprising that different patterns of changes in forest structure and function with altitude have been reported. Körner [23] pointed out that "there are two categories of environmental changes with altitude: those physically tied to meters above sea level, such as atmospheric pressure, temperature and clear-sky turbidity; and those that are not generally altitude specific, such as moisture, hours of sunshine, wind, season length, geology and even human land use." Forest presence in the first instance is determined by limits of temperature and precipitation [17]. Given sufficient conditions of temperature and precipitation, forest structure and process are shaped by local microclimatic conditions, disturbance frequency and history, edaphic conditions, and a variety of anthropogenic influences [24], [25], [26]

From this perspective, we do not expect generality in comparing tropical elevational transects. Over any significant elevational range temperature will always decrease. How that decrease affects forest structure and function however will vary idiosyncratically among elevational transects depending on the factors mentioned above. Tree diameter growth rates in particular, and hence stand-level dynamics in old-growth forest, will be strongly determined by precipitation and insolation. A transect that spans relatively hot wet lowland sites to cool dry montane sites will likely be very different from a transect from hot dry lowland sites to cool moist montane sites. And a transect such as the Volcán Barva transect, which is humid to perhumid at every site with moderate temperatures, would be yet again different, even without considering conditions of disturbance, edaphic factors and human influences.

The most interesting questions for ecosystem science on any given transect are therefore "What are the relative roles of climate, soil, disturbance, and human influence on the physical and biological structure of forests on this transect?" Resolving some of these issues, for example the relative roles of different nutrients in limiting productivity, can only be addressed through experimental manipulations [27]. Disturbance frequency and intensity is a major driver of ecosystem structure and function, and both are hard to quantify with scattered plots measured infrequently [28], [29].

At this point in our research we can summarize the Volcán Barva Transect (VBT) in relation to other tropical elevational transects as follows:

1. The VBT is completely humid to perhumid; lack of water is not a major restriction on tree growth, although excess water and associated cloudiness likely is.
2. Species diversity is moderate by continental tropical rain forests standards [30], and follows the common pattern of a peak in species diversity at elevations above the lowest elevations sites, with a steep drop-off in arboreal species at higher elevations. Trees form the bulk of biodiversity of stems ≥10 cm diameter at all elevations.
3. Forest physical structure is characterized by (a) a low frequency at all elevations of canopy gaps extending to the forest floor ("Brokaw gaps" [21]), (b) taller lowland canopies with scattered emergents grading to shorter and more homogeneous canopies at higher elevations, and (c) intermediate levels of stand density and basal area in the non-cloud-forest sites.
4. Stem number and plot basal area, as well as rates of diameter growth, mortality and recruitment are generally intermediate by tropical rain forest standards [30].
5. Forest above-ground biomass residence time increases substantially with elevation.
6. The sections of the landscape selected for study (cf S1 File) were not obviously affected by physical disturbances at spatial scales close to our plot size (1 ha) or larger for at least the last several decades, and direct recent human impacts of extraction were not evident with the exception of understory palms at higher elevations.
7. Forest soils are well developed and extensive at every site, and non-soil microsites such as rock outcrops account for minimal area.

In summary, the VBT plot network is characterized by a large elevational gradient with closed-canopy forest at all elevations, rugged terrain, high rainfall and forest soil coverage, intermediate levels of species diversity, dynamics and tree diameter growth rates, and minimal direct impacts of human intervention. In the following section we address how this description applies to the larger Volcán Barva landscape outside the plots, as well as a variety of issues related to elevational transect studies using permanent forest inventory plots.

### Issues facing forest ecology projects on tropical elevational transects

As we designed and implemented the network of forestry inventory plots along this tropical elevational transect we faced a number of theoretical and practical issues. While similar research efforts on other tropical elevational transects (5], [9], [10], [11], [12], [13], [14], [15] have faced identical issues, these have not been thoroughly identified and discussed. Here we briefly present these issues and discuss our efforts to address them or suggest their implications for future research.

#### Biased Sampling

Globally tropical mountain landscapes are characterized by steep terrain, with an estimated 75% of the planimetric area of tropical montane forest occurring on slopes of >27* [9]. Many of these slopes exceedingly difficult or practically-speaking impossible to work on (Fig 2). Like most researchers in this situation [9], we located our plots on the flattest areas available, and avoided areas like recent landslides, streams or rivers within the 1-ha plots, or significant areas of non-tree vegetation like bamboo patches. In addition our sites were sufficiently drained so that they were not permanently or seasonally inundated. We made conscious decisions to sample a restricted portion of the landscape, and we went to significant efforts to systematize our site selection process to avoid additional biases like the majestic tree effect within the flat sites we selected (see S1 File for plot placement criteria). Given the resources available, we believe these were justifiable decisions. We were however aware from the beginning that our sites might represent areas with the highest biomass and perhaps the lowest turnover rates on this landscape. While our data could be scaled from plot to landscape, we currently have no way of knowing how accurate such generalizations would be.

Two studies indicate the magnitude of the potential bias involved in extrapolating from small plots. Spracklen and Righelato [9] found that the mean slope angle of tropical mountain forests worldwide was 32%, with 75% of the area on slopes of >27%; in contrast, the mean slope angle of 45 forest inventory plots was only 17%. In a pioneering study using lidar to compare forest characteristics of tropical montane forest landscapes and forest inventory plots on these landscapes, Marvin et al. [31] showed that scaling results from single 1-ha plots usually lead to moderately to severely biased estimates of landscape-scale forest canopy structure and biomass.

A major challenge for future research on tropical elevational transects on rugged terrain is identifying strategies for scaling from plots to landscapes. This will clearly involve remote sensing [31], although the two major technologies for direct remote sensing estimates of biomass, radar and lidar, both have significant issues on steep terrain. Some type of distributed sampling strategy is called for, so that the complete range of vegetation types and densities is covered, at least with one-time sampling. The most powerful data will be those combining as nearly concurrently as possible remote sensing with terrestrial measurements. From both the terrestrial and remote sensing side it will be difficult to sample the steep and highly dynamic portions of these landscapes, which is of course the main reason there are few data from these areas. Accurate representation of the landscapes will require that we move beyond our current flat-plot biased vision and develop ways of sampling the entire range of slopes, aspects, soils and disturbance regimes characteristic of tropical mountainous areas.

A separate sampling issue is replication and plot size: for a given amount of money, what field design gives the most information? There is no easy answer to this question but it still should be asked [32]. Very small plots are highly sensitive to normal tree-fall disturbance events. Typically a plot size of 0.25–1 ha is sufficient to dampen that effect [33]. But the effects of sampling less frequent larger-scale disturbances are harder to determine and have been the source of controversy [28], [29]. We chose to maximize sampling of the elevational gradient at the expense of estimating within-elevation variance in forest structure and process. Had the elevational gradient been 500 m instead of 2700 m, we might have chosen to replicate within elevations. An additional reason for few but larger plots was that we were also using these plots as ground data for remote sensing studies with radar and lidar. Larger plots present fewer issues for georegistering ground and remotely sensed data. Ultimately the science goals of a project should drive decisions on replication and plot size, but what gets put on the ground will be a compromise between the ideal design and the realities of logistics and funding.

A related issue is sampling frequency. Common practice for large forest plots is to census multi-year intervals, frequently five years or more [30]. Climate however varies both predictably and stochastically on annual time scales. Forest responses to annual climate variation can become impossible to detect with forests are measured on multi-annual time intervals [34], [35]. Because much of the interest in forests on tropical elevational transects has to do with forests' relation to climate, there is a strong argument for sampling at annual or sub-annual time steps. Detecting old-growth forest responses to the normal variation of climate factors requires a sufficient run to data to isolate signal from noise [35]. There are few data sets for tropical forests with even five consecutive annual remeasurements, and there are very few data for old-growth forest plots with >10 consecutive annual remeasurements (cf the TEAM Project at Conservation International and [22]). With so few cases of synchronized data on annual performance and climate, it is not surprising that there is no consensus of the effects of climate change on tropical forest performance. The paucity of this type of data underscores the importance of the few attempts we know of to create long-term annual-time step tropical forest data sets, such as the TEAM project, the Center for Tropical Forest Science and the CARBONO Project in Costa Rica [36].

#### Missing climate data

Tropical mountain elevational transects generally involve steep and sometimes non-linear gradients in climate. This presents major challenges for obtaining accurate climate data at the plot scale. Two major climatic drivers affecting forest productivity and dynamics are temperature and rainfall [22]. Of the two, temperature is far more tractable to monitor for at least two reasons. Except in exceptional circumstances and over restricted elevational ranges, temperature decreases in a predictable fashion with elevation (cf Table 1). Additionally, temperature can be monitored at low cost using field dataloggers like the HOBO sensors we used. Precipitation however bears no necessary relation with elevation [23], and can vary significantly on the scale of several kilometers. On the Volcán Barva transect for example rainfall almost doubles over the 17 km between the La Selva met station at 50 m elevation and the rainfall gauge at the Rara Avis Ecolodge at 900 m elevation (Table 1). Rainfall can also be sensitive to local geographic conditions. Without rainfall data at the plot scale, it is not possible to assess the accuracy of data from weather stations several to dozens of kilometers from the plot, or of using data from global databases scaled to local conditions. To date there is no technology for measuring rainfall comparable to the microdataloggers used for temperature monitoring. Automated rain gauges are an order of magnitude more expensive and require constant maintenance in tropical forest conditions. Another significant limitation is that accurate rainfall measurements require either an above-canopy platform or a large open clear space, both of which can be problems at isolated field sites in rugged forested terrain.

Good data on site-specific insolation are also hard to obtain and are particularly important on elevational transects, since the location of persistent clouds can be a major factor limiting insolation. Similar to the situation with rainfall however, there are not yet cheap low-maintenance radiation dataloggers, and measuring insolation also requires a large open area or above canopy-access.

Future research should include an emphasis on site-based climate monitoring. Site-based climate data are critical for a detailed understanding of powerful drivers of forest productivity and dynamics [22]. Site-based data also provide the ultimate reality check on alternant approaches such as using estimates of rainfall, temperature and insolation from global data sets derived from satellite data.

#### Carbon stocks estimation

Many studies of tropical rain forests, including those on elevational transects, report data on above-ground biomass. These data are frequently and incorrectly reported as measurements of biomass, whereas in fact they are estimates of unknown accuracy based on allometric relations of trunk diameter and height to biomass [19]. The problems outlined by Clark and Kellner [19] are accentuated on elevational transects for several reasons. Wood density is frequently inversely proportional to growth rate [37]. We have very little data on the relation of species' growth rate to elevation (see following section), so how or if the majority of species show wood density shifts with elevation is generally not known. Forest height frequently decreases with elevation, but whether single-species allometries shift is generally not known. Problems of the allometry of multi-stemmed trees can be ignored in the lowlands but became significant with elevation in our study area (Table 2). In this research we opted to report direct measurements (diameter and the conversion to basal area) rather than biomass estimates of unknown and currently unknowable accuracy. Progress on obtaining biomass estimates of known accuracy will require substantial research including plot harvest on the spatial scale of remote sensing data [19], which in our case was not feasible logistically, politically or financially.

#### Species-level research on elevational transects

The current distribution and performance of individual species can be used to derive predictions of forest changes in response to future climate scenarios, particularly global temperature increases [38]. Among the 355 species and morphospecies sampled in this study, 21 were restricted to one or both of the highest elevation plots (2400 and 2800 m). Given the observed rate of change of temperature with altitude (~0.54* C per 100 m elevation Table 1), an increase of regional temperature of 2–3* C. would put all of these species outside their observed thermal ranges on this transect. It seems highly likely many or most of these species will go extinct on this transect under most current projections of global temperature increases [39]. The disproportionate mortality of highland versus lowland species in this transect has already been detected [40]. A similar situation exists with the lowest plots, where 34 species were observed only in the 100 and/or 200 m plot. A 1* rise in temperature would put these species outside their observed thermal ranges on this transect. In contrast to the high-elevation species however, these lowland species could potentially migrate upslope [1], [3].

There are many ecological questions that do not require extensive knowledge of individual species, but there are a series of pressing questions about the effects of global climate change on tropical rain forests that require knowledge of species' distributions and performance. The average tropical trees species in the lowlands occurs at low densities [30], [41], so all

tropical forest studies face severe problems trying to accumulate adequate sample size for species-level studies. This is particularly so for demographic rates like recruitment and mortality, which as previously mentioned typically have much larger coefficients of variation than growth rates. For tropical transect studies the sample-size problem is exacerbated in the lower elevations where species diversity is highest, and within elevation replication may be sacrificed for better sampling along the elevational gradient. At higher elevations this situation is somewhat alleviated since species diversity is much lower. At the 100 m plot for example only 11% of the species had sample sizes of N≥10 individuals, whereas at the 2800 m the figure was 45%.

For tree species that reach the canopy, improvements in remote sensing including higher spectral and spatial resolution satellite data and the increasing capabilities of Unmanned Aerial Vehicles will soon lead to better estimates of species' distributions and canopy-level rates of mortality, recruitment and crown area dynamics [42], [43], [44]. There are not yet similar technical solutions in view for better estimates of the distribution and performance of subcanopy individuals.

## Conclusions

Global climate change will inexorably and on ecological time scales rapidly impact tropical rain forest structure and function globally [22], [39]. Given their steep environmental gradients, the effects of changing temperature and rainfall on forests are expected to be most quickly observed on elevational transects, and a growing literature supports this expectation [6], [7], [40]. With so few transects established in species-rich tropical forest, it is important to maintain the existing research efforts and establish additional sites. Greatly expanding spatial coverage will require incorporating remote sensing of different types, but ground plots will be needed to provide key data necessary to validate, calibrate, interpret and supplement remotely-sensed data. Documenting the patterns of extinction, migration, productivity and dynamics will take sustained study on the order of decades. At the moment all the tropical elevational studies we are aware of are sustained by short-term grants. There is a pressing need for national or international funding that will recognize tropical elevational transects as semi-permanent research sites similar to the U.S. National Science Foundation's Long-Term Ecological Research and Long-Term Environmental Research projects. Tropical elevational transect networks can be front-line observatories for the pace of forest change in the face of global climate change. Realizing this research potential, however, will require new approaches to securing and maintaining funding over the decadal scales necessary to document forest changes with statistically powerful time series.

## Supporting Information

S1 File Protocols for siting the 1-ha forest inventory plots. (PDF)

S1 Dataset Dataset for canopy height maps at all sites. (TXT)

S2 Dataset Dataset for crown heights at all sites. (TXT)
