# Supplementary material for: Tropical Rain Forest Structure, Tree Growth and Dynamics along a 2700-m Elevational Transect in Costa Rica
Source: PLoS One. 2015 Apr 9;10(4):e0122905. doi: 10.1371/journal.pone.0122905 (PMC4391938; doi:10.1371/journal.pone.0122905)
Supplement: S1 File — (PDF) [file pone.0122905.s001.pdf]

Protocol for siting free-standing 1-ha vegetation plots -- D.B. Clark, November 2009 (followed in the Volcán Barva Transect TEAM Project and also adopted for Clark/Saatchi NASA Braulio plots)

Purpose of one-ha vegetation plots.

The one-ha vegetation plots are designed to provide representative samples of old-growth forest to enable long-term study of forest composition and performance, stratified across the gradients of interest in the larger landscape studied by a TEAM project.

Protocol design issues:

For the purpose of this protocol the principal gradients affecting forest structure over the landscape studied by a given TEAM project must be identified by the Vegetation Scientist. These will frequently be edaphic-hydrologic, degree of slope, temperature, rainfall, and natural and anthropogenic disturbance. It will not be possible to replicate across all of these gradients, so *a priori* decisions must be made as to the principal gradients to be sampled.

All sources consulted (Condit 1998, Dallmeir et al. 1992, TEAM 2006, Rainfor 2007) agreed it was desirable to select an area of relatively homogenous soil and vegetation of a type that represents the dominant soil and vegetation (in areal terms) of the larger landscape.

With 1-ha plots It is also necessary to take care not to bias site selection by seeking areas of especially open and tall forest (the “majestic forest” bias, Rainfor 2007).

Specific design criteria for this protocol:

Building on these and other recommendations of these authors, the following specific design criteria were developed for the Volcán Barva Transect TEAM Project free-standing 1-ha vegetation plots.

1. Plots will be sited in a stratified random design. The exact starting point will be determined in a random fashion (see below).
2. The principal gradients to be sampled will be defined *a priori*. In the case of the Volcán Barva project the principal gradient to be studied will be elevation. We will therefore try to minimize variation along all other gradients, in particular be selecting relatively flat sites on homogenous soil in old growth that contain no streams and associated riparian vegetation.
3. Sites will be in old growth with at least 200 m buffer from areas judged to be secondary (see below) and at least 50 m and therefore out of sight of any frequented trail.
4. Among equally promising areas to be searched for potential plot sites, sites closer to the base camp will be preferred.
5. For this protocol “flat” will have to be determined subjectively in the beginning until a better idea of the magnitude of slopes at this spatial scale in the new study areas can be obtained. For the moment we’re defining “flat” as “no more slope than currently existing 1-ha vegetation plots”.

Field Protocol:

1. Use all available remotely sensed and cartographic data to assess the local area prior to plot establishment. Take waterproof maps with satellite land use history, topography, and gridded UTM locations into the field.

2. First examine the general area (say within 1 hour of the base camp) to determine if there are obvious differences in land use history, soils, local natural disturbance (wind throw in particular) and slope. Sample soils with a soil probe during this reconnaissance to get a general idea of variation in soil color and texture. Decide what type of vegetation, slope and soil conditions

represent the target conditions for the 1-ha vegetation plot, and document this with notes and photographs.

3. Pick the closest likely-looking old-growth area. Randomly (use a true random method like having someone pick 1 or 2 while another person has decided which way each one means) pick left or right of the path, unless one of these directions is blocked for some reason, in which case go to the other side. Another valid reason to pick one direction over the other is if the maps suggest a potential problem on one side but not the other. Document the choice if it is not strictly random.

4. Determine the direction perpendicular to the trail (nearest 10*) and walk in that direction, using a compass and measuring tape, 50.0 m. At exactly 50.0 m stop and mark this point with a stake, this is the mid-point (50:0) of the plot edge, which should be roughly parallel to nearest the trail. If a stream is visible in what would be the plot area but the area otherwise looks suitable, move a predetermined distance (30, 40, 50 m etc) in the reference direction to reach a new base point.

5. Choose the plot's exact baseline orientation based on staying parallel to the slope as estimated at this point, and/or avoiding natural obstacles visible from this point such as streams or cliffs.

6. Examine the area ahead to determine if there is any reason to reject the site at this time. Only the following factors are allowable reasons: slope excessive (unwalkable or greater than the subjectively-determine criteria for "flat"), stream flowing inside the plot area, obvious soil discontinuity or vegetation obviously secondary or disturbed by humans. Criteria for secondary or disturbed vegetation include vegetation changes (like stem diameter distributions) along straight lines, a high prevalence of multiple-fork trees, sawn logs, old fences or other structures, or obvious secondary vegetation (grasses, large patches secondary species such as Cecropiaceae etc.). Because the goal is to measure tree growth and diversity, sites with >25% non-tree vegetation like bamboo will be rejected. On the other hand, sites that are old growth should have at least a few natural treefall gaps at the 1-ha scale. If no gaps at all are seen this suggests mature secondary forest. Document each time a site is rejected with a clear explanation of the reason.

7. If the plot is not suitable but appears that it could be made suitable by advancing or shifting left or right, shift the entire plot to fit the landscape. Only the circumstances listed above justify such as shift, moving to avoid natural gaps or to include particularly easy-to-measure sections must be strictly avoided. Document the reason for each shift.

8. Once a starting point is found, walk the boundaries and center line of the plot using a tape and compass. Look into the potential plot area and see if there is any reason to reject this plot based on current evidence. If there are and they can be avoided, shift the 50:0 point by 5-10 m increments in the plot-axis directions (parallel to the slopes). Document the reason for each shift.

9. Continue sampling soil with a soil corer, looking for major discontinuities in color or texture, and continue looking for influences of secondary vegetation or human impacts throughout this process. Major soil discontinuities (e.g. deep soil into hardpan, loam into mottled waterlogged profiles) are reasons to abandon a given site. Document soil and vegetation appearance.

10. Once the outline of the plot is completed, begin surveying with surveying transit. First establish the 50:0 to 50:100 baseline, then survey 50 m segments perpendicular to this to get the grid intersections, from every 10 m along the baseline.

References:

Condit, R.. 1998. *Tropical forest census plots: methods and results from Barro Colorado Island, Panama and a comparison with other plots*, New York:Springer-Verlag, 1998. 211 pages.

Dallmeier, F., M. Kabel, and R. Rice. 1992. "Methods for long-term biodiversity inventory plots in protected tropical forests." *Long-term monitoring of biological diversity in tropical forest areas. Methods for establishment and inventory of permanent plots.* (Dallmeier, F., Ed.). UNESCO. Paris. pp 11-46.

TEAM Vegetation Protocol (June 2006 version)

RAINFOR field manual. 2007. October 2007 Web download.
